# Diagnostic Dilemma: Tuberculosis Mastoiditis in an Immunocompromised Patient

**DOI:** 10.7759/cureus.65797

**Published:** 2024-07-30

**Authors:** Siew Lee Tiong, Wan Nurul Aida, Razak I, Shahrul Hitam

**Affiliations:** 1 Otolaryngology - Head and Neck Surgery, Hospital Ampang, Ampang, MYS

**Keywords:** immunocompromised patient, acid fast bacilli (afb), unilateral facial nerve palsy, painless otorrhoea, mtb protein chain reaction (pcr), tuberculosis mastoiditis

## Abstract

Tuberculosis (TB) is endemic in Malaysia but rarely affects the middle ear cleft. Common presentations of TB mastoiditis include unilateral, painless otorrhea, multiple small perforations of the tympanic membrane, and facial nerve palsy, although these symptoms can vary among patients. The diagnosis of TB mastoiditis is challenging due to its rarity and its similar presentation to common bacterial ear infections. This often leads to missed diagnoses, resulting in significant delays in treatment and potential complications. CT scans and histopathological examinations are crucial for diagnosing TB mastoiditis. Real-time polymerase chain reaction offers higher sensitivity and specificity compared to conventional methods for detecting *Mycobacterium tuberculosis*. TB infection should be considered in cases of otitis media that do not respond well to empirical antibiotic therapy. It is essential to send appropriate samples for TB testing to ensure timely diagnosis and treatment. This case report highlights the diagnostic challenges and complications encountered in a 22-year-old immunocompromised woman with TB mastoiditis.

## Introduction

Tuberculosis (TB) is a widespread disease primarily caused by* Mycobacterium tuberculosis*, which is known for causing pneumonia. While *M. tuberculosis* is a significant healthcare concern, extrapulmonary TB accounts for only 15-20% of all cases and rarely affects the middle ear cleft, representing only 0.05-0.9% of chronic otitis media cases [1,2]. The tubercular bacillus can invade the temporal bone through several routes, including aspiration via the Eustachian tube, hematogenous spread from distant sites, or contiguous spread from nearby extracranial or intracranial infections [1,3]. Diagnosing TB mastoiditis is challenging as it often mimics common bacterial ear infections. CT scans typically reveal soft tissue density in the middle ear and mastoid without specific evidence of bone erosion, making it nonspecific for TB mastoiditis. Therefore, an accurate diagnosis usually requires a histopathological examination (HPE) or microbiological culture [1]. The initiation of anti-TB therapy is often delayed in such cases. This report presents the diagnostic challenges and complications encountered in a 22-year-old immunocompromised woman with TB mastoiditis.

## Case presentation

**Figure 1:**
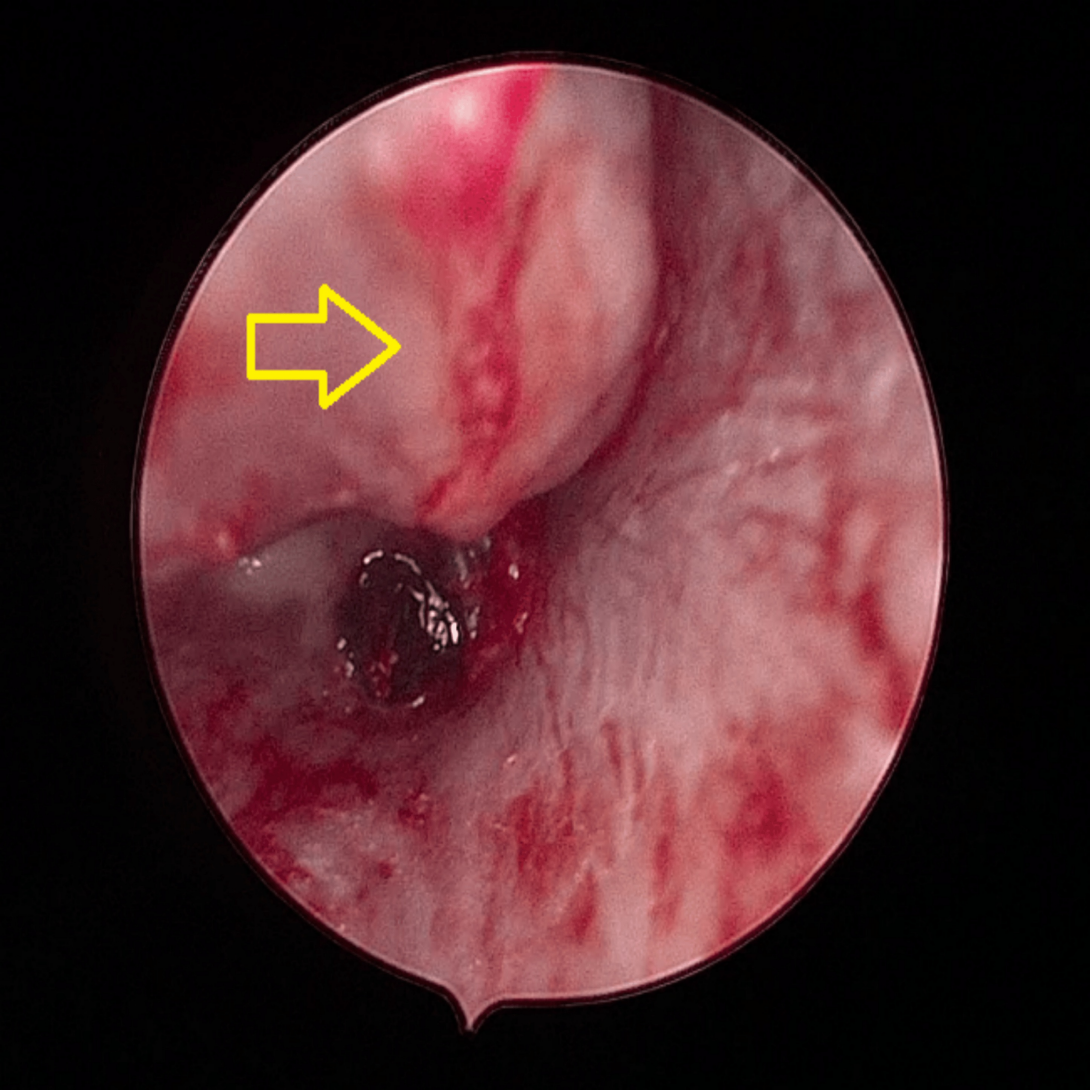
Otoendoscopy showed posterior sagging of the canal wall with granulation tissue extending from the middle ear

We present the case of a 22-year-old woman with underlying systemic lupus erythematosus (SLE) complicated by lupus nephritis, serositis, hematological and mucocutaneous manifestations, probable neuropsychiatric SLE, end-stage renal failure, a psychotic disorder, and a history of empiric treatment for smear-negative pulmonary TB with oral Akurit-4 for four months two years prior. She presented with a two-week history of right otorrhea and otalgia. Otoendoscopy revealed mucopurulent discharge with keratin debris and granulation tissue in the right external auditory canal (EAC), with the tympanic membrane obscured. A diagnosis of acute otitis media with cholesteatoma was made, and she was started on topical ear drops and oral Augmentin. Unfortunately, she missed the follow-up and returned one month later with right facial nerve palsy, House-Brackmann (HB) Grade IV. Repeated otoendoscopy showed posterior sagging of the posterior canal wall with granulation tissue from the middle ear (Figure 1).

**Figure 2:**
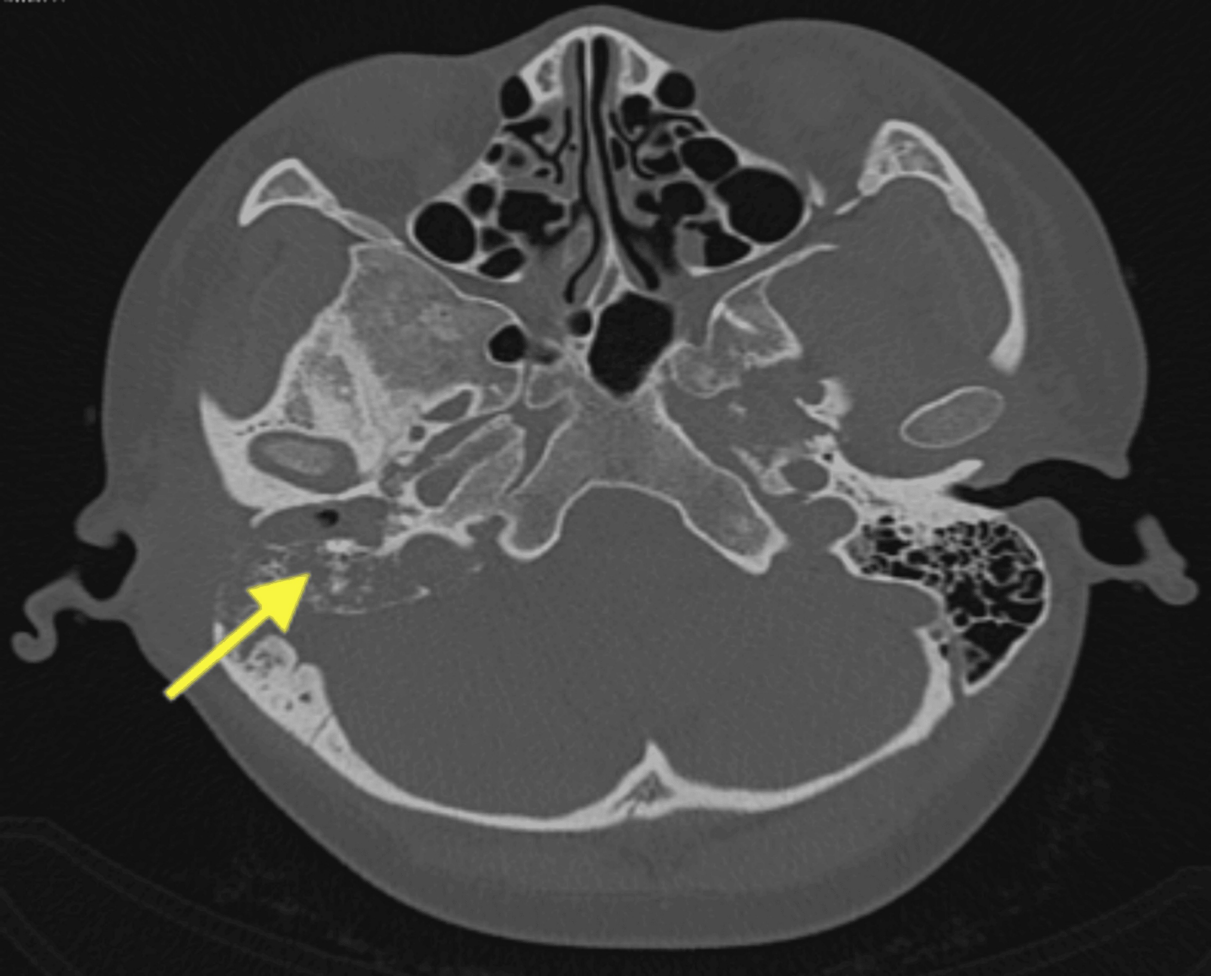
HRCT temporal bone scan showed right acute otitis media and mastoiditis with extensive bony erosion HRCT, high-resolution CT

Hearing assessment revealed unilateral right moderate to profound mixed hearing loss. A high-resolution CT (HRCT) temporal bone scan showed right acute otitis media and mastoiditis, complicated by subperiosteal and right temporal epidural abscess, right temporal meningitis, and right sigmoid sinus thrombosis (Figure 2).

A multidisciplinary team discussion was conducted. Medical management was chosen due to the high risk of surgery associated with her underlying comorbidities, and she strongly refused surgical intervention. Pus samples from the right EAC were sent for culture, which showed *Pseudomonas aeruginosa*. However, the acid-fast bacilli (AFB) smear was negative. She was started on intravenous cefepime and meropenem for one month, but a repeated HRCT temporal bone scan showed no improvement, with only a minimal reduction of about 1 cm² in the subperiosteal abscess.

Diagnosing the patient proved challenging due to the lack of disease improvement despite antibiotic treatment. Laboratory tests from previous admissions were reviewed, and all tuberculous tests, including mycobacterium C+S from skin samples, multiple sputum AFB smears, mycobacterium C+S, and *M. tuberculosis* polymerase chain reaction (PCR) from CSF, showed negative results. Additionally, the patient reported no chronic cough, night sweats, fever with chills, recent travel history, or close contact with tuberculosis. The patient eventually consented to aspiration of the right subperiosteal abscess through a postauricular approach, and the specimens were sent for laboratory testing. While both the AFB smear and TB C+S tests yielded negative results, the diagnosis of tuberculosis was confirmed only after the TB PCR test showed a positive result six weeks later.

**Figure 3:**
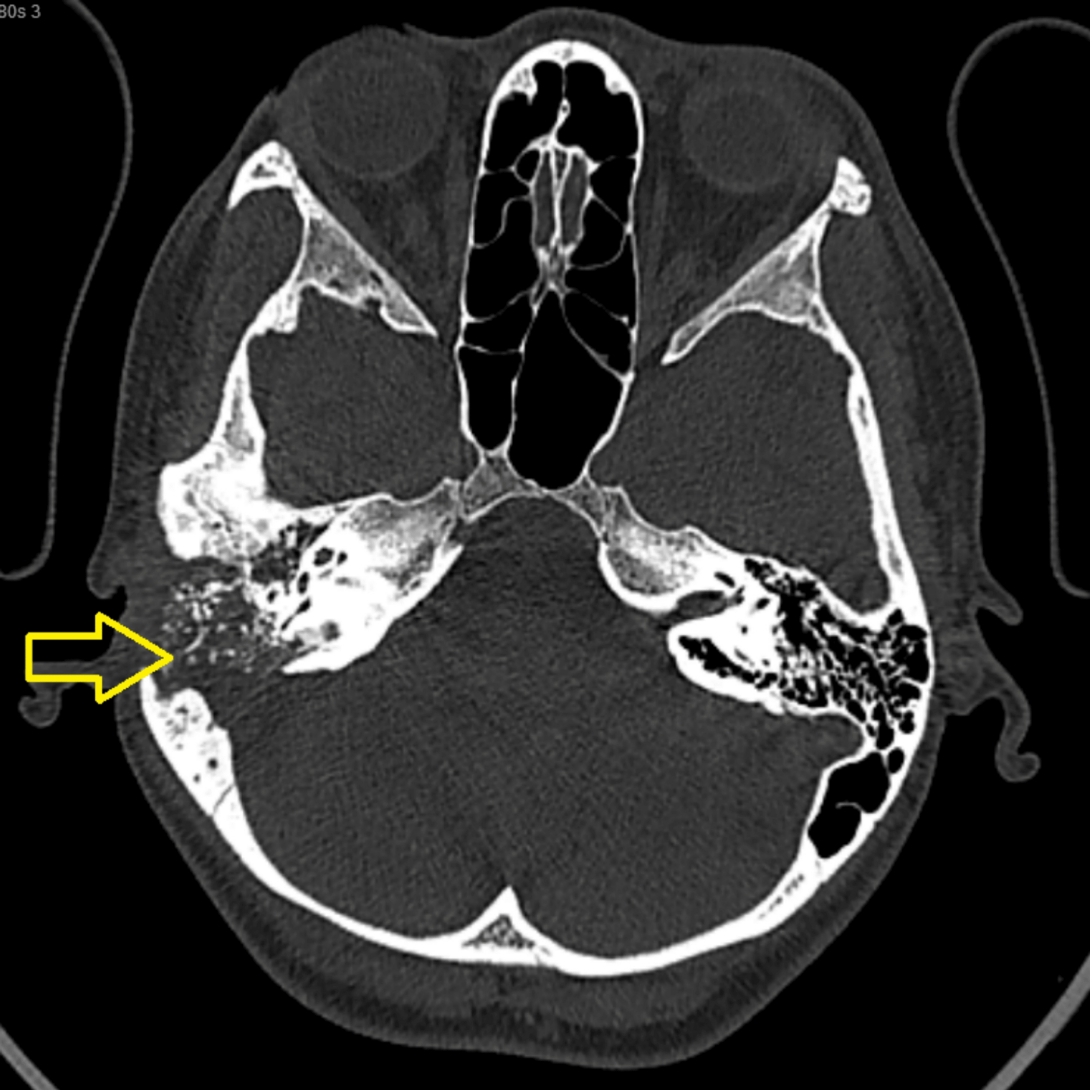
Repeated contrast-enhanced CT brain showed resolved right-sided subperiosteal mastoid collection six months post-anti-TB medication

Intensive anti-TB treatment (ATT) with streptomycin, ethambutol, isoniazid, rifampin, and pyrazinamide was administered for two months, followed by maintenance treatment with isoniazid and rifampin (HR) for seven months. A contrast-enhanced CT brain scan was repeated after six months of ATT, which showed the resolution of the right-sided subperiosteal mastoid collection and adjacent sigmoid sinus thrombosis (Figure 3). The patient has been on ATT for six months. The otorrhea subsided, but the facial nerve function remained static at HB Grade IV.

## Discussion

Globally, TB is a chronic bacterial infection caused by *M. tuberculosis*, a slow-growing, strict aerobic bacillus that forms granulomas with caseous necrosis in response to the affected tissue. TB mastoiditis is a rare form of extrapulmonary TB, with a prevalence estimated to be less than 0.1%, and it more commonly affects children than adults [1,2].

The symptoms of TB of the middle ear can vary based on the patient’s immunological status. Wallner described the typical symptoms in 1953 as painless otorrhea, multiple perforations of the tympanic membrane, pale granulation tissue, ipsilateral peripheral facial palsies, early severe hearing loss, and bone necrosis [4]. However, these classic presentations may have evolved over time and now exhibit a more pleomorphic range. A comprehensive review by Skolnik et al. of all reported cases of TB middle ear infections in the English literature found that facial palsy is present in only 16% of cases, and multiple tympanic perforations are equally rare [5].

In the case described, the patient initially presented with otalgia and otorrhea, which later progressed to include ipsilateral facial nerve palsy, mastoiditis, subperiosteal abscess, right temporal epidural abscess, right temporal meningitis, and right sigmoid sinus thrombosis one month later. Complications frequently arise when diagnosis is delayed. Sens et al. proposed that the prognosis heavily depends on prompt diagnosis and early treatment rather than decompression. Complete recovery is more likely if treatment begins within five days of the onset of paralysis; however, if treatment is delayed for more than two months, recovery may not occur [6]. Unfortunately, due to the delay in treatment, our patient experienced complications, including ongoing facial nerve palsy, despite the resolution of the subperiosteal abscess and adjacent sigmoid sinus thrombosis.

The HRCT of the temporal bone in our patient revealed extensive bony erosion and right temporal meningitis. Similar aggressive disease progression has been documented in several case reports. Cavallin and Muren proposed the diagnosis of TB otomastoiditis when CT scans show extensive bone destruction without clinical signs of an aggressive infection [7]. Intracranial invasion is uncommon because the dura mater typically prevents the spread of infection; when it does occur, it is often due to dissemination through the bloodstream [6].

MacAdam and Rubio reported a case of slowly developing hearing loss, indicating that hearing loss in a TB middle ear infection can be variable [8]. In the early phase, hearing assessment typically reveals a conductive pattern due to the accumulation of exudate in the middle ear and the formation of granulation tissue. As the inflammatory process extends, it can lead to necrosis and erosion of the ossicular chain, otic capsule, and mastoid cells, resulting in mixed hearing loss or sensorineural hearing loss [8].

On the other hand, mycobacterial counts in extrapulmonary TB are low, making AFB cultures rare. Additionally, demonstrating AFB from ear discharge is challenging due to the presence of additional infections. Bacteriological examination of ear discharge is often unreliable, as the growth of* M. tuberculosis *can be obscured by other organisms such as *Staphylococcus*, *Pseudomonas*, *Klebsiella*, *Proteus*, and *Streptococcus *[9]. In our patient’s case, the right ear pus swab culture and sensitivity revealed growth of *P. aeruginosa*, while the AFB smear and TB culture were negative. Garg et al. reported that real-time PCR (RT-PCR) has higher sensitivity for detecting *M. tuberculosis*, finding that MTB was detected by RT-PCR in 28.6% of cases where AFB smear results were negative [10].

## Conclusions

While TB is common, TB mastoiditis is rare. TB should be suspected in cases of otitis media that do not respond well to empirical antibiotic therapy. Untreated or delayed treatment of TB mastoiditis can lead to severe, permanent complications, such as facial palsy, hearing impairment, and intracranial spread of the infection. CT scans and HPEs are crucial for diagnosing TB mastoiditis. RT-PCR offers higher sensitivity and specificity compared to conventional methods. Early detection and treatment of TB mastoiditis are essential, particularly in highly endemic regions, among immunocompromised patients, or in individuals with a history of or suspicion of TB disease.
